# Beyond surgical showcase: workflow continuity and exploratory AI-oriented video interpretability of robotic low anterior resection videos on YouTube

**DOI:** 10.3389/fsurg.2026.1886815

**Published:** 2026-07-22

**Authors:** Adem Ozcan, Gizem Gunes

**Affiliations:** Department of Surgical Oncology, Ankara Bilkent City Hospital, Ankara, Türkiye

**Keywords:** AI-oriented interpretability, robotic colorectal surgery, robotic low anterior resection, robotic rectal surgery, surgical education, surgical video analysis, total mesorectal excision, YouTube

## Abstract

**Background:**

Publicly available robotic surgical videos are increasingly used as supplementary educational resources in colorectal surgery. However, beyond general educational quality, the extent to which robotic low anterior resection (LAR) videos preserve workflow continuity, critical anatomical visualization, and expert-rated AI-oriented video interpretability remains unclear. This study aimed to evaluate publicly available robotic LAR videos on YouTube for workflow completeness, educational continuity, and exploratory AI-oriented video interpretability.

**Methods:**

A structured cross-sectional video-based analysis of publicly available robotic LAR videos on YouTube was performed using predefined robotic rectal surgery-related search terms. Workflow domains including setup phases, vascular dissection, pelvic dissection, total mesorectal excision (TME), critical anatomical visualization, reconstruction phases, and safety-oriented procedural steps were systematically evaluated. Workflow completeness and AI-oriented video interpretability were assessed using structured expert-rated frameworks, whereas educational value was summarized using an author-derived exploratory composite score. Associations between video characteristics and composite scores were analyzed.

**Results:**

Thirty-nine robotic LAR videos met the eligibility criteria. Median video duration was 647 s. Total mesorectal excision (TME) dissection, pelvic dissection, distal rectal transection, and inferior mesenteric artery/inferior mesenteric vein (IMA/IMV) dissection were among the most consistently demonstrated operative phases. In contrast, patient positioning, trocar placement, robotic docking, specimen extraction, and leak testing demonstrated substantially lower representation rates. Dunn's *post-hoc* comparisons with Bonferroni correction demonstrated higher workflow completeness, educational value, and AI-oriented interpretability scores in structured workflow videos than in highlight/showcase videos. Workflow analysis demonstrated marked heterogeneity across operative domains, with pelvic dissection-related phases showing the highest visibility rates. Viewer engagement metrics were not significantly associated with workflow completeness or AI-oriented interpretability.

**Conclusions:**

Publicly available robotic low anterior resection videos on YouTube predominantly emphasize visually prominent pelvic dissection phases while inconsistently preserving complete operative workflow continuity. Many robotic LAR videos may function more as selective technical demonstrations than as standardized workflow-oriented educational resources. These findings should be interpreted as video-level workflow and interpretability observations, not as evidence of effects on trainee performance, operative quality, patient safety, or actual AI model performance. Future robotic surgical video platforms may benefit from workflow-oriented reporting standards emphasizing procedural continuity, anatomical interpretability, reconstruction, and safety-oriented workflow representation.

## Graphical Abstract

**Figure:**
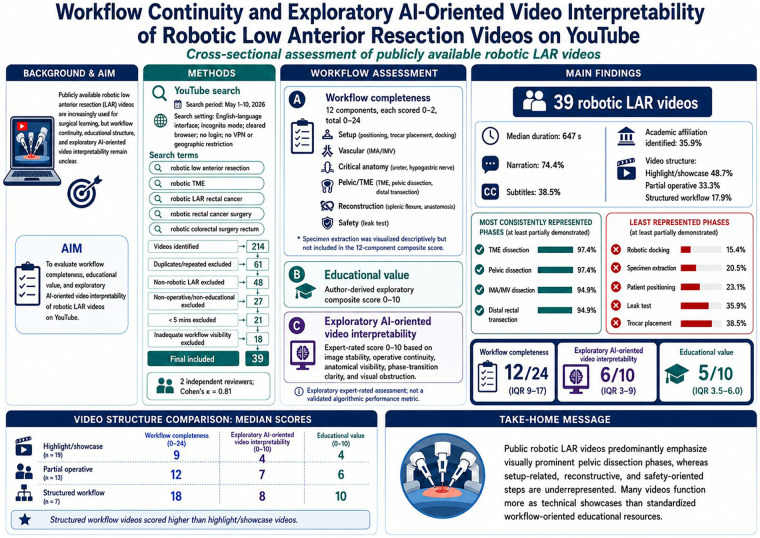

## Introduction

Robot-assisted colorectal surgery, particularly robotic low anterior resection (LAR), has become an increasingly established approach in rectal cancer surgery because of its technical advantages in the narrow pelvis, including stable visualization, improved dexterity, and enhanced ergonomics (1, 2). In parallel with this technical evolution, surgical video has become an important component of modern operative education. Publicly accessible platforms such as YouTube are now widely used by surgical trainees and practicing surgeons as supplementary resources for procedural learning and operative preparation (3–5).

Previous YouTube-based studies have mainly evaluated general reliability, viewer engagement, and conventional educational quality metrics (5–9, 26, 27). Although such assessments are useful, they may not adequately capture what matters most in robotic rectal surgery: whether the video preserves the operative workflow in a way that allows the viewer to understand the sequence, anatomy, and technical logic of the procedure. For robotic LAR, the educational value of a video depends not only on image quality or narration, but also on the structured demonstration of key operative phases, including setup, vascular pedicle control, total mesorectal excision (TME), critical anatomical visualization, reconstruction, and safety-related steps (10, 11).

This question has become more relevant with the emergence of surgical artificial intelligence (AI), computer vision, and workflow recognition systems. Surgical videos are no longer only passive educational material; they are increasingly considered procedural data sources for phase recognition, workflow segmentation, anatomical identification, skill assessment, and automated annotation (12–17). In this context, publicly available videos may be evaluated for workflow-oriented interpretability, although such assessment should be distinguished from validation of actual computational model performance. For these applications, videos require more than visually impressive operative footage. They require temporal continuity, interpretable phase transitions, clear anatomical exposure, and limited editing-related disruption (11, 14–17).

Despite the growing availability of robotic surgical videos online, it remains unclear whether publicly available robotic LAR videos provide structured workflow-oriented educational content or mainly present selected visually compelling operative segments. This distinction is important for both contemporary surgical education and the future use of public operative videos in AI-oriented workflow analysis. Therefore, the present study aimed to evaluate robotic LAR videos available on YouTube using a structured expert-derived framework focused on workflow completeness, educational value, critical anatomical visualization, and exploratory AI-oriented video interpretability.

## Materials and methods

### Study design

This cross-sectional observational study evaluated the workflow continuity, educational structure, and exploratory AI-oriented video interpretability of publicly available robotic low anterior resection (LAR) videos on YouTube. The study specifically focused on how robotic rectal surgery workflows were represented within publicly accessible operative videos and whether these videos preserved sufficient procedural continuity for educational interpretation and future workflow-oriented surgical video analysis.

The study design was conceptually informed by previous literature regarding surgical workflow recognition, surgical video annotation, AI-assisted surgical education, robotic surgical video analysis, and workflow-oriented surgical video assessment principles, including LAP-VEGaS-related educational assessment concepts (10–18, 28). Because all analyzed videos were publicly accessible and did not contain identifiable patient information, formal institutional review board approval was not required.

### Video search strategy

A structured YouTube search was performed between May 1 and May 10, 2026, using predefined robotic colorectal surgery-related search terms. Searches were conducted using an English-language YouTube interface in incognito mode, without user login, and after clearing browser history and cache to minimize personalization. No VPN, manual geographic restriction, or location filter was applied. YouTube's default relevance-based search output order was used for screening. View counts, like counts, and comment counts were recorded on May 10, 2026.

The following search terms were used:

“robotic low anterior resection”

“robotic TME”

“robotic LAR rectal cancer”

“robotic rectal cancer surgery”

“robotic colorectal surgery rectum”

Approximately the first 30–40 results were screened for “robotic low anterior resection” and “robotic TME”, and the first 20–30 results were screened for the remaining search terms, consistent with the search log provided in Supplementary Table 1. Video screening continued until no additional eligible workflow-oriented robotic LAR videos were identified within consecutive search output screening. Duplicate videos, repeated uploads, and non-eligible videos were excluded during the screening process. Videos were screened sequentially according to YouTube default relevance-based search output order. A supplementary video-level dataset is provided as Supplementary Dataset 1. It includes the public YouTube URL, video title at the time of screening, channel category, upload date and year, duration, engagement metrics recorded on May 10, 2026, narration, subtitle availability, academic affiliation, video structure category, component-level workflow scores, workflow completeness score, AI-oriented video interpretability domain and composite scores, and the author-derived educational value score. An accompanying codebook specifies all variable definitions, coding rules, recoding procedures, and composite-score derivations. The dataset does not include downloaded video files, screenshots, thumbnails, reproduced video frames, or patient-identifiable information.

### Eligibility criteria

Videos were included if they:

1. demonstrated robotic low anterior resection or robotic rectal cancer resection,
2. contained intraoperative robotic surgical footage,
3. allowed evaluation of operative workflow phases,
4. had sufficient visual quality for anatomical and workflow assessment,
5. and had a duration of at least 5 min.

The 5-min minimum duration threshold was selected to exclude very short promotional clips or isolated operative fragments that were unlikely to provide sufficient sequential intraoperative footage for phase-level workflow assessment. Videos were not excluded solely because of non-English narration if the intraoperative footage was visually interpretable and allowed assessment of predefined workflow components. Narration and subtitle availability were recorded as separate educational features.

Videos were excluded if they:

- consisted solely of promotional or commercial content,
- contained only isolated edited fragments without sufficient sequential intraoperative footage for phase-level workflow assessment,
- lacked meaningful operative footage,
- represented duplicate uploads,
- involved non-colorectal robotic procedures,
- or had inadequate visual quality preventing workflow analysis.

When multiple uploads of the same operative video were identified, only the most comprehensive version was included. For clarity, excluded isolated edited fragments were distinct from eligible highlight/showcase videos. Videos categorized as highlight/showcase were condensed or selectively edited but still contained assessable intraoperative robotic LAR footage allowing evaluation of predefined workflow components. In contrast, excluded edited fragments consisted only of isolated clips without sufficient operative sequence or workflow continuity for scoring.

### Video assessment and extracted variables

All videos were independently evaluated by two reviewers experienced in robotic colorectal surgery. Overall interobserver agreement for workflow-oriented video assessment was evaluated using Cohen's kappa coefficient. Discrepancies were resolved through consensus review before final score generation. Interobserver agreement demonstrated substantial consistency (Cohen's *κ* = 0.81). The reported *κ* value represented an overall exploratory estimate of reviewer agreement across workflow-oriented video assessments rather than construct-specific psychometric validation.

For each video, the following variables were recorded:

- upload year,
- video duration,
- number of views,
- number of likes,
- number of comments,
- narration presence,
- subtitle availability,
- channel type,
- academic affiliation,
- disease indication,
- robotic platform,
- and video structure category.

Videos were classified according to structural presentation as:

- highlight/showcase videos,
- partial operative videos,
- or structured workflow videos.

Highlight/showcase videos were defined as eligible condensed operative videos that emphasized selected technical segments but retained sufficient intraoperative footage for scoring of predefined workflow components. Partial operative videos were defined as videos showing multiple sequential operative phases but not the full procedural workflow. Structured workflow videos were defined as videos preserving a more continuous operative sequence across major procedural domains, including setup, dissection, reconstruction, and/or safety-related steps.

### Workflow completeness score

A composite workflow completeness score was developed to quantify the extent to which essential robotic LAR workflow phases were represented within each video.

The score included the following operative domains:

- patient positioning,
- trocar placement,
- robotic docking,
- inferior mesenteric artery/inferior mesenteric vein (IMA/IMV) dissection,
- ureter visualization,
- hypogastric nerve visualization,
- splenic flexure mobilization,
- TME dissection,
- pelvic dissection,
- distal rectal transection,
- anastomosis,
- and leak testing.

Each workflow component was graded using a predefined 3-point scale:

- 0 = not demonstrated,
- 1 = partially demonstrated,
- 2 = clearly demonstrated.

The composite workflow completeness score ranged from 0 to 24, with higher scores representing more complete procedural workflow representation. Specimen extraction was analyzed descriptively as a workflow-related component in Table 2 and Figure 2 but was not included in the predefined 12-component composite workflow completeness score.

### Exploratory AI-oriented video interpretability assessment

An exploratory AI-oriented video interpretability assessment was generated to evaluate expert-rated video characteristics potentially relevant to workflow-oriented surgical video analysis and future annotation tasks. This assessment was not designed to measure or predict the performance of any computational workflow-recognition, computer vision, or automated annotation model.

The exploratory AI-oriented video interpretability assessment domain included assessment of:

- image stability,
- operative field continuity,
- critical anatomical visibility,
- operative phase transition clarity,
- smoke/blood/blur-related visual obstruction,

Each parameter was scored as:

- 0 = poor,
- 1 = moderate,
- 2 = good.

The exploratory AI-oriented video interpretability score ranged from 0 to 10, with higher scores indicating greater expert-rated workflow continuity, anatomical interpretability, phase-transition clarity, and visual consistency.

### Educational value score

Educational value was summarized using an author-derived exploratory composite score based on five video-level components: narration, subtitle availability, procedural comprehensibility, critical anatomical visibility, and operative field continuity.

Narration was coded as 0 when absent and 2 when present, and subtitle availability was coded as 0 when absent and 2 when present. Procedural comprehensibility was approximated using the structural presentation category and was coded as 0 for highlight/showcase videos, 1 for partial operative videos, and 2 for structured workflow videos. Critical anatomical visibility and operative field continuity were each scored as 0 (poor), 1 (moderate), or 2 (good), consistent with the corresponding expert-rated video interpretability domains.

The five component scores were summed to generate an educational value score ranging from 0 to 10, with higher scores indicating greater educational structure and procedural comprehensibility. Because structural presentation category contributed directly to the procedural comprehensibility component, and because critical anatomical visibility and operative field continuity overlapped with the AI-oriented video interpretability framework, comparisons of educational value across video structure categories were considered exploratory and subject to partial conceptual circularity. The educational value score should not be interpreted as a validated educational outcome instrument.

### Operational definitions

To improve scoring consistency, predefined operational definitions were established before video assessment.

- **Clearly demonstrated:** operative step or anatomical structure was continuously visible and educationally interpretable.
- **Partially demonstrated:** operative component was identifiable but incompletely visualized or briefly presented.
- **Not demonstrated:** operative component was absent or not assessable.

Critical anatomical visualization required recognizable exposure of relevant structures, including the ureter or hypogastric nerves, with sufficient anatomical continuity for educational interpretation.

Operative phase transition clarity referred to the ability to identify progression between major workflow phases without excessive fragmentation or editing-related discontinuity.

The exploratory AI-oriented video interpretability assessment used in the present study was an expert-derived observational framework and was not validated against a computational workflow-recognition algorithm, computer vision model, or automated annotation system.

### Statistical analysis

Statistical analyses were performed using IBM SPSS Statistics for Windows, Version 22.0 (IBM Corp., Armonk, NY, USA). Continuous variables were expressed as median and interquartile range (IQR) because of non-normal data distribution. Categorical variables were summarized as frequencies and percentages.

Comparisons between video structure groups were performed using the Kruskal–Wallis test for continuous or ordinal variables. Because structural presentation category contributed to the procedural comprehensibility component of the educational value score, comparisons of educational value across video structure groups were treated as exploratory and were not interpreted as independent validation of the educational value construct. When omnibus Kruskal–Wallis testing demonstrated statistical significance, Dunn's *post-hoc* pairwise comparisons with Bonferroni correction were performed, and exact adjusted *p*-values were reported. Effect size for Kruskal–Wallis analyses was summarized using epsilon-squared (*ε*^2^). Pairwise non-parametric effect sizes were summarized using Cliff's delta where appropriate.

Spearman correlation analysis was used to evaluate associations between ordinal video structure category and workflow completeness and AI-oriented video interpretability scores. The association between video structure category and educational value was not interpreted as an independent correlation because structural presentation category contributed directly to the procedural comprehensibility component of the educational value score. Because of the limited sample size, particularly the small number of structured workflow videos, subgroup analyses were considered exploratory. Potential confounding by video duration, upload year, narration, subtitle availability, academic affiliation, and channel type was considered when interpreting subgroup comparisons; however, multivariable modeling was not performed because of the limited number of included videos and the small structured workflow subgroup.

A two-sided *p*-value <0.05 was considered statistically significant.

## Results

**Figure 1:**
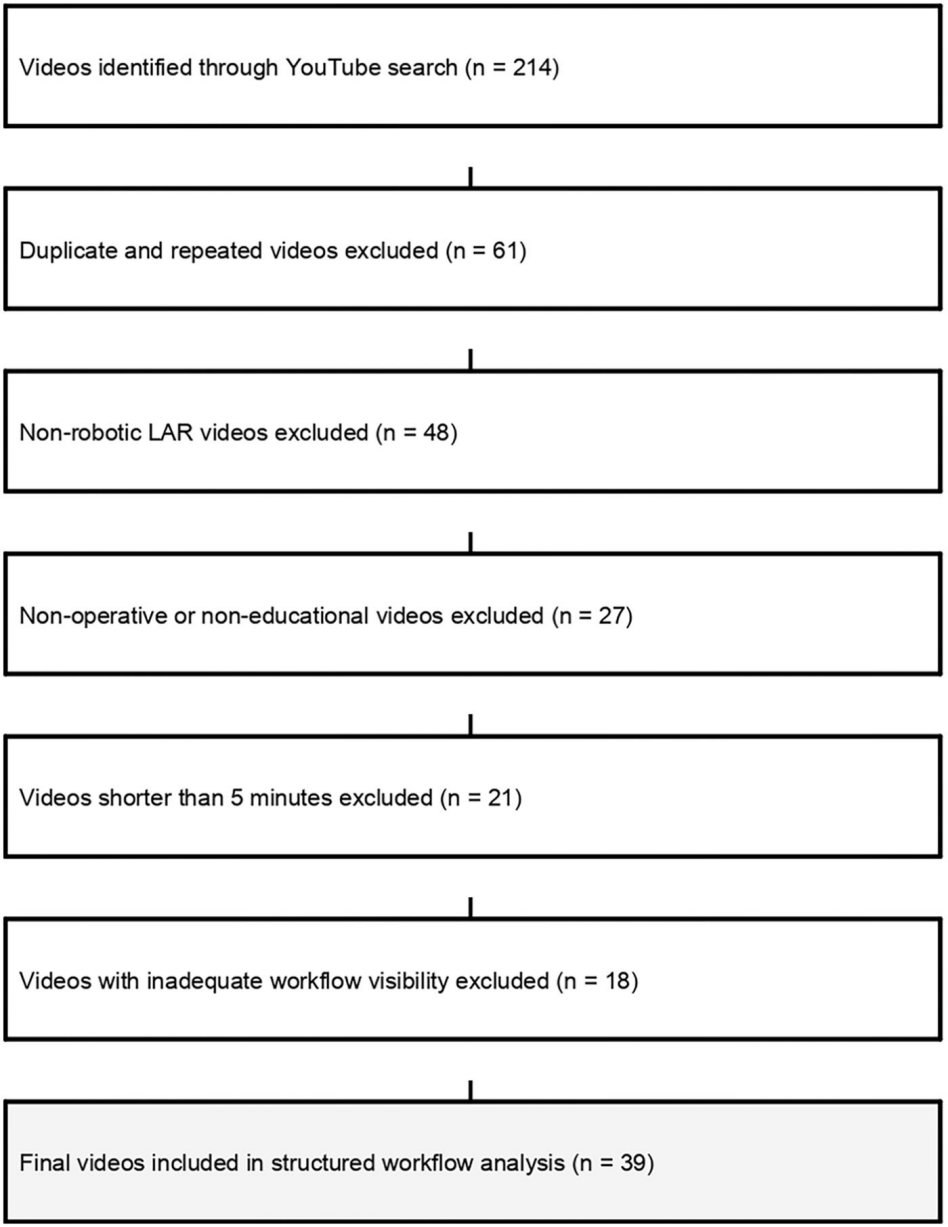
Flow diagram of video identification, screening, eligibility assessment, and final inclusion of robotic low anterior resection videos analyzed in the study. Searches were performed using an English-language YouTube interface in incognito mode with a cleared browser and without login-based personalization.

A total of 39 publicly available robotic low anterior resection (LAR) videos met the eligibility criteria and were included in the final analysis (Figure 1). The videos were uploaded between 2013 and May 2026, with a median upload year of 2021 (IQR: 2018–2023). Median video duration was 647 s (IQR: 577–942), corresponding to approximately 10.8 min. The median view count was 1,833 (IQR: 790–8,148), whereas median like and comment counts were 23 (IQR: 9–63) and 1 (IQR: 0–4), respectively (Table 1).

**Table 1:** Baseline characteristics of included robotic Low anterior resection videos.

Variable	Value
Total videos	39
Video metrics	
Upload year, median (IQR)	2021 (2018–2023)
Upload year, range	2013–May 2026
Video duration, seconds, median (IQR)	647 (577–942)
View count, median (IQR)	1,833 (790–8,148)
Like count, median (IQR)	23 (9–63)
Comment count, median (IQR)	1 (0–4)
Educational features	
Narration present, *n* (%)	29 (74.4)
Subtitles available, *n* (%)	15 (38.5)
Academic affiliation identified, *n* (%)	14 (35.9)
Video structure	
Highlight/showcase videos, *n* (%)	19 (48.7)
Partial operative videos, *n* (%)	13 (33.3)
Structured workflow videos, *n* (%)	7 (17.9)
Channel type	
Individual surgeon, *n* (%)	30 (76.9)
Journal-based educational platform, *n* (%)	7 (17.9)
Academic institution, *n* (%)	2 (5.1)
Disease type	
Malignant disease, *n* (%)	26 (66.7)
Benign disease, *n* (%)	2 (5.1)
Unspecified disease indication, *n* (%)	11 (28.2)
Primary dissection direction	
Medial-to-lateral, *n* (%)	35 (89.7)
Lateral-to-medial, *n* (%)	3 (7.7)
Hybrid/combined, *n* (%)	1 (2.6)
Robotic platform	
Xi, *n* (%)	3 (7.7)
Si, *n* (%)	2 (5.1)
SP, *n* (%)	1 (2.6)
Unspecified platform, *n* (%)	33 (84.6)

IQR, interquartile range.

Most videos originated from individual surgeon channels (76.9%), whereas 17.9% were associated with journal-based educational platforms and 5.1% with academic institutions. Malignant disease was the predominant indication (66.7%). Narration was present in 74.4% of the videos, while subtitles were available in 38.5%. Based on structural classification, 48.7% were categorized as highlight/showcase videos, 33.3% as partial operative videos, and 17.9% as structured workflow videos (Table 1).

**Figure 2:**
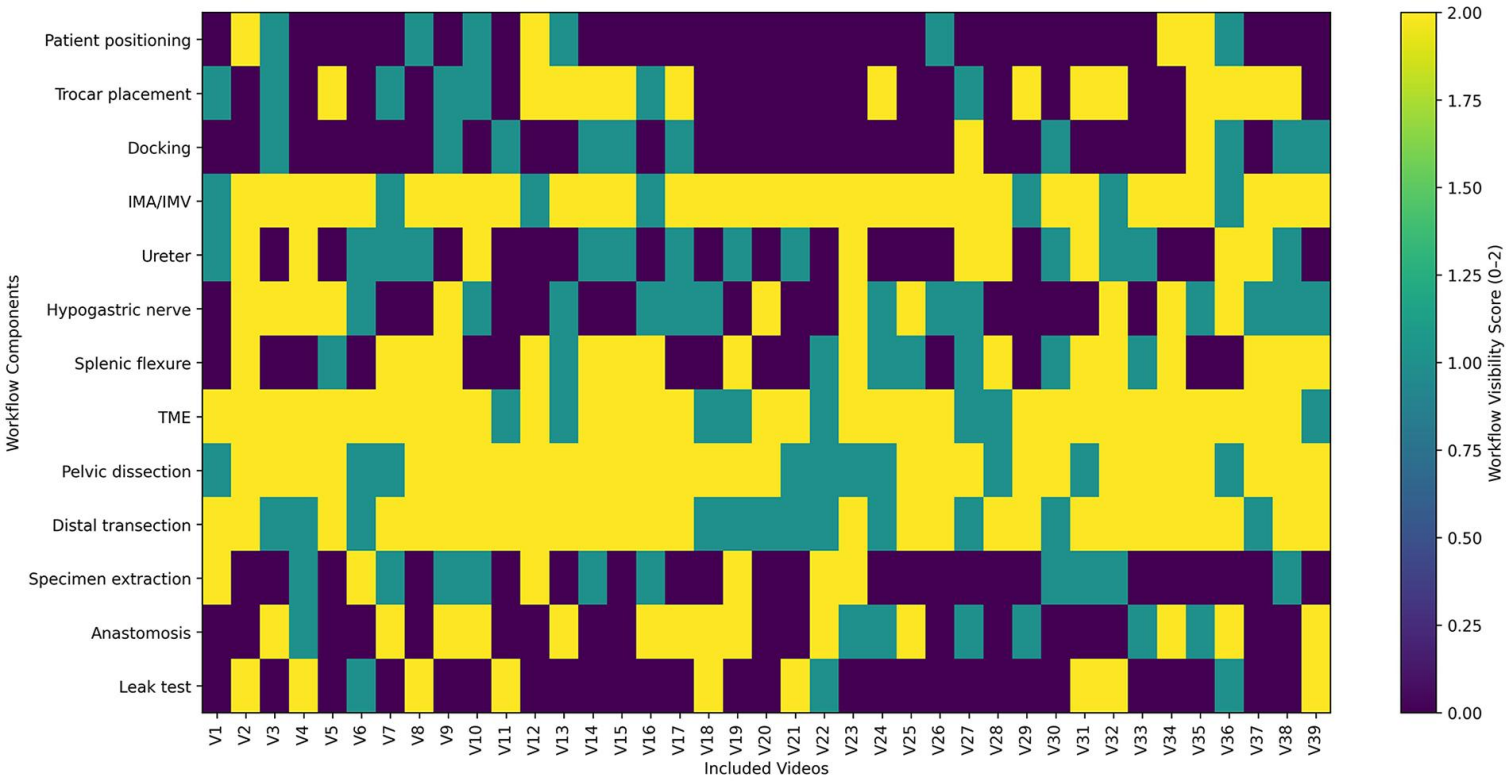
Workflow completeness heatmap demonstrating the distribution and visibility of operative workflow components across included robotic low anterior resection videos. Workflow components were graded as 0 (not demonstrated), 1 (partially demonstrated), or 2 (clearly demonstrated). Specimen extraction was visualized descriptively but was not included in the 12-component composite workflow completeness score.

Marked variability was observed across operative workflow components when at least partial demonstration was considered (Table 2, Figure 2). Total mesorectal excision (TME) dissection and pelvic dissection were among the most consistently represented phases, being at least partially visible in 97.4% of videos. Distal rectal transection and inferior mesenteric artery/inferior mesenteric vein (IMA/IMV) dissection were identified in 94.9% of videos. In contrast, patient positioning was visible in 23.1% of videos, trocar placement in 38.5%, and robotic docking strategy in 15.4%. Specimen extraction and leak testing were present in 20.5% and 35.9% of videos, respectively.

**Table 2:** Visualization of operative workflow components across included robotic low anterior resection videos, including At least partial demonstration.

Workflow component	Not demonstrated *n* (%)	Partially demonstrated *n* (%)	Clearly demonstrated *n* (%)	At least partially demonstrated *n* (%)
Patient positioning	30 (76.9)	5 (12.8)	4 (10.3)	9 (23.1)
Trocar placement	24 (61.5)	7 (17.9)	8 (20.5)	15 (38.5)
Robotic docking strategy	33 (84.6)	3 (7.7)	3 (7.7)	6 (15.4)
IMA/IMV dissection	2 (5.1)	8 (20.5)	29 (74.4)	37 (94.9)
Ureter identification	12 (30.8)	10 (25.6)	17 (43.6)	27 (69.2)
Hypogastric nerve visualization	17 (43.6)	9 (23.1)	13 (33.3)	22 (56.4)
Splenic flexure mobilization	26 (66.7)	5 (12.8)	8 (20.5)	13 (33.3)
TME dissection	1 (2.6)	5 (12.8)	33 (84.6)	38 (97.4)
Pelvic dissection	1 (2.6)	4 (10.3)	34 (87.2)	38 (97.4)
Distal rectal transection	2 (5.1)	4 (10.3)	33 (84.6)	37 (94.9)
Specimen extraction	31 (79.5)	3 (7.7)	5 (12.8)	8 (20.5)
Anastomosis	11 (28.2)	9 (23.1)	19 (48.7)	28 (71.8)
Leak test	25 (64.1)	0 (0.0)	14 (35.9)	14 (35.9)

IMA, inferior mesenteric artery; IMV, inferior mesenteric vein; TME, total mesorectal excision.

Critical anatomical visualization also varied across videos. Ureter identification was clearly or partially visible in 69.2% of videos, whereas hypogastric nerve visualization was present in 56.4%. Splenic flexure mobilization was clearly represented in 20.5% of videos (Table 2).

**Figure 3:**
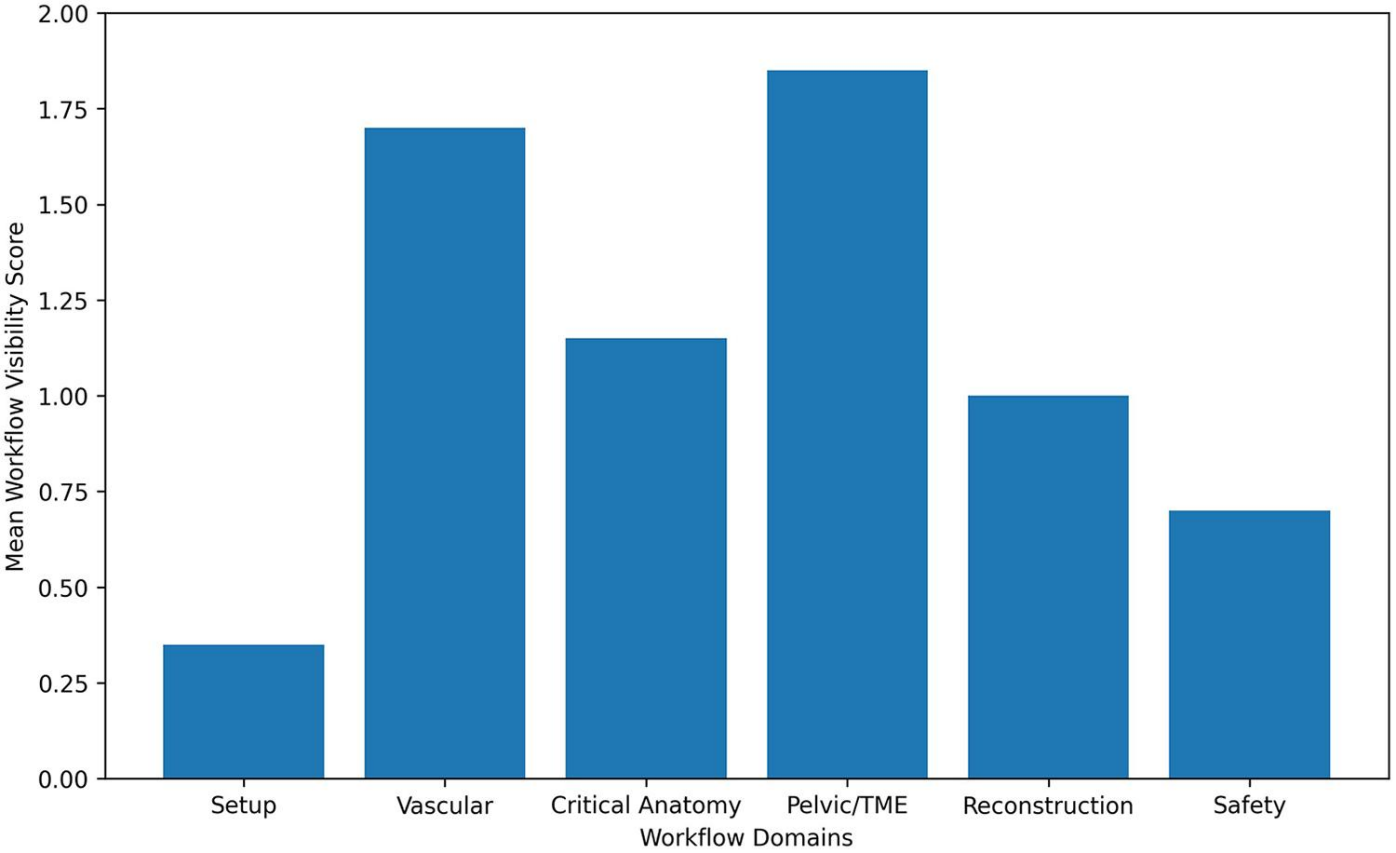
Distribution of operative workflow domains across included robotic low anterior resection videos. Pelvic/TME and vascular phases demonstrated the highest workflow visibility scores, whereas setup- and safety-related domains showed substantially lower representation. Domain categories were used for descriptive visualization of workflow representation and were not intended to represent validated procedural quality domains.

Workflow domain analysis demonstrated lower representation rates within setup- and safety-related domains compared with pelvic dissection-related domains (Figure 3). The workflow domain categorization used for Figure 3 is summarized in Supplementary Table 2. Pelvic/TME-related components showed the highest workflow visibility scores, whereas setup-related components showed the lowest representation rates.

The median workflow completeness score was 12/24 (IQR: 9–17), the median exploratory AI-oriented video interpretability score was 6/10 (IQR: 3–9), and the median educational value score was 5/10 (IQR: 3.5–6.0) (Table 3). Workflow completeness differed significantly across video structure categories, with median scores of 18, 12, and 9 for structured workflow, partial operative, and highlight/showcase videos, respectively (Kruskal–Wallis *H* = 9.17, *p* = 0.010; *ε*^2^ = 0.20). Dunn's *post-hoc* pairwise comparisons with Bonferroni correction showed that structured workflow videos had higher workflow completeness scores than highlight/showcase videos (adjusted *p* = 0.008; Cliff's *δ* = 0.71), whereas the differences between highlight/showcase and partial operative videos (adjusted *p* = 0.727) and between partial operative and structured workflow videos (adjusted *p* = 0.153) were not statistically significant.

**Table 3:** Workflow completeness, educational value, and exploratory AI-oriented video interpretability according to video structure.

Video type	n (%)	Workflow completeness score median (IQR)	AI-oriented ınterpretability score median (IQR)	Educational value score median (IQR)
Highlight/showcase videos	19 (48.7)	9 (7–11)	4 (2–5)	4 (3–5)
Partial operative videos	13 (33.3)	12 (10–15)	7 (4–7)	6 (5–6)
Structured workflow videos	7 (17.9)	18 (17–21)	8 (8–9)	10 (8–10)
*p*-value		**0.010**	**0.001**	**<0.001**

Comparisons between groups were performed using the Kruskal–Wallis test followed by Dunn's *post-hoc* pairwise comparisons with Bonferroni correction when appropriate. Exact adjusted *p*-values are reported in the Results text. IQR, interquartile range.

Bold values indicate statistically significant *p*-values (*p* < 0.05).

Median AI-oriented interpretability scores were 8, 7, and 4 for structured workflow, partial operative, and highlight/showcase videos, respectively (Kruskal–Wallis *p* = 0.001; *ε*^2^ = 0.31). Dunn's *post-hoc* pairwise comparisons showed higher AI-oriented interpretability in structured workflow videos than in highlight/showcase videos (adjusted *p* = 0.001; Cliff's *δ* = 0.82). The comparison between partial operative and structured workflow videos did not remain statistically significant after Bonferroni correction (adjusted *p* = 0.064), and the comparison between highlight/showcase and partial operative videos was not significant (adjusted *p* = 0.422). Median educational value scores were 10, 6, and 4 for structured workflow, partial operative, and highlight/showcase videos, respectively (Kruskal–Wallis *H* = 17.98, *p* < 0.001; *ε*^2^ = 0.44). Dunn's *post-hoc* pairwise comparisons with Bonferroni correction showed higher educational value scores in structured workflow videos than in highlight/showcase videos (adjusted *p* = 0.00012; Cliff's *δ* = 0.92). Differences between highlight/showcase and partial operative videos (adjusted *p* = 0.05382) and between partial operative and structured workflow videos (adjusted *p* = 0.12044) were not statistically significant after Bonferroni correction. Given the small number of structured workflow videos, all subgroup comparisons were interpreted as exploratory.

No significant association was identified between total view count and workflow completeness or exploratory AI-oriented video interpretability assessment. Similarly, upload year showed no significant relationship with workflow completeness, educational value, or exploratory AI-oriented video interpretability assessment.

Spearman correlation analysis demonstrated positive associations between ordinal video structure category and workflow completeness (rho = 0.459, *p* = 0.003) and AI-oriented video interpretability (rho = 0.622, *p* < 0.001). These associations were interpreted descriptively because video structure classification conceptually overlapped with workflow continuity-related scoring domains.

**Figure 4:**
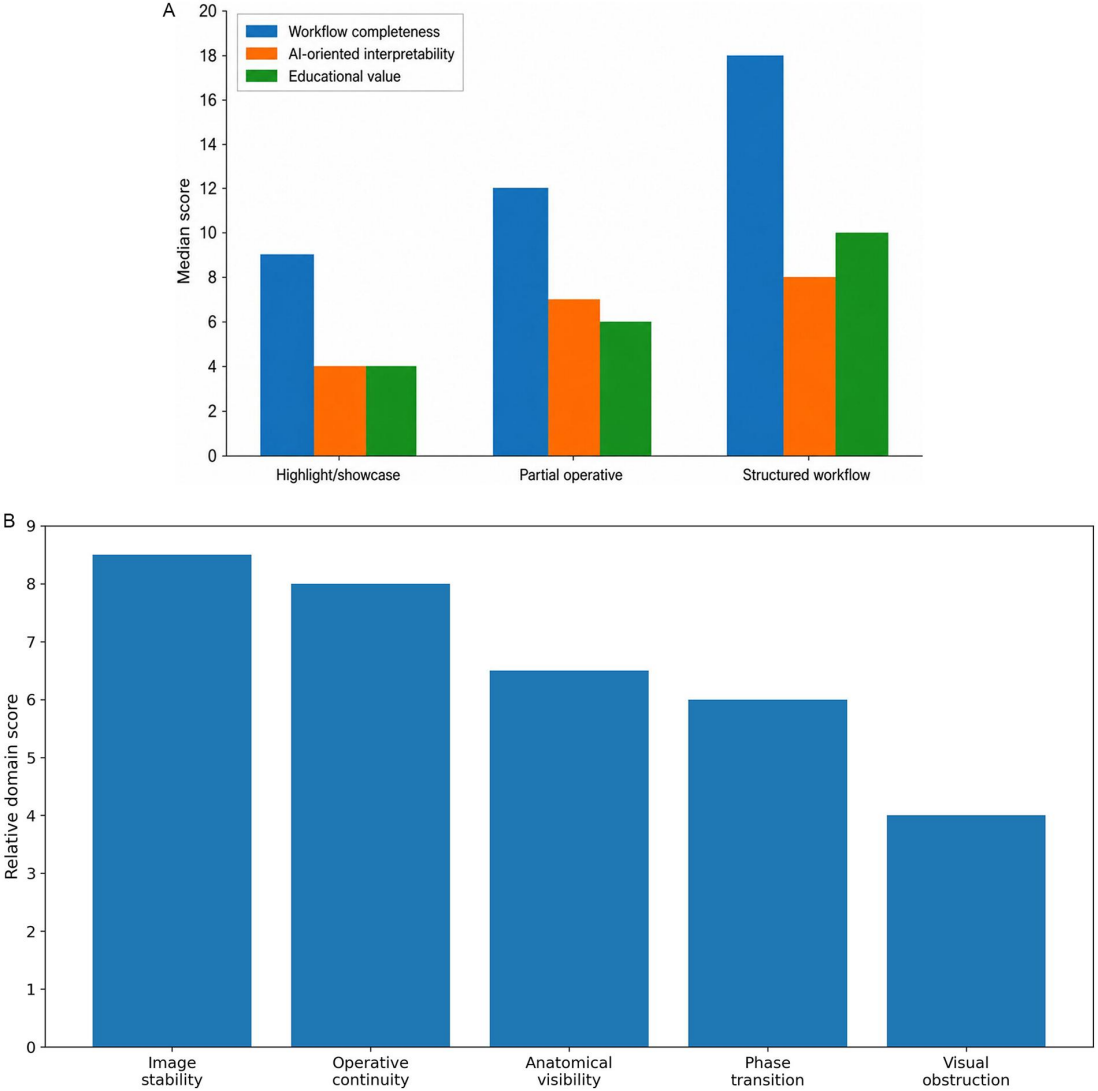
**(A)** Comparison of workflow completeness, educational value, and exploratory AI-oriented video interpretability scores according to video structure categories. Scores are presented as median values for highlight/showcase, partial operative, and structured workflow videos. **(B)** Distribution of exploratory AI-oriented video interpretability domains across included robotic low anterior resection videos. Image stability and operative continuity demonstrated the highest relative domain scores, whereas visual obstruction-related domains demonstrated comparatively lower scores.

Exploratory AI-oriented video interpretability domain analysis demonstrated variability in image stability, operative continuity, anatomical visibility, phase-transition clarity, and visual obstruction across videos (Figure 4B). Videos with preserved operative continuity and stable visualization tended to show higher AI-oriented interpretability scores.

## Discussion

The present study demonstrated substantial heterogeneity in workflow continuity, educational structure, and exploratory AI-oriented video interpretability among publicly available robotic low anterior resection videos on YouTube. Although pelvic dissection-related phases such as TME, pelvic dissection, and distal rectal transection were consistently represented, setup-related, reconstructive, and safety-oriented workflow components were demonstrated less frequently. These findings indicate that publicly available robotic LAR videos do not uniformly preserve complete procedural workflow representation.

Previous YouTube-based surgical studies have primarily focused on reliability, educational quality, and engagement metrics (5–7). However, robotic colorectal surgery possesses unique workflow characteristics that may not be adequately reflected by conventional video quality assessments. Robotic rectal surgery is inherently phase-dependent and relies heavily on sequential procedural continuity, stable visualization, and anatomical orientation (1, 2). Accordingly, workflow preservation may represent a more technically relevant measure of educational utility in robotic surgery.

One of the principal findings of the present study was the imbalance between highly represented pelvic dissection phases and underrepresented setup-related workflow domains. TME and pelvic dissection demonstrated the highest visibility rates, whereas patient positioning, trocar placement, docking, specimen extraction, and leak testing were less consistently demonstrated. Similar concerns regarding selective operative representation and edited surgical video culture have previously been discussed in the context of surgical education and surgical data science (19, 20).

Structured workflow videos demonstrated higher workflow completeness, educational value, and AI-oriented interpretability than highlight/showcase videos. This finding is relevant in light of recent developments in surgical workflow recognition and computer vision-based operative analysis. Contemporary AI-assisted surgical systems increasingly depend on temporally coherent and anatomically interpretable operative videos for workflow segmentation and automated annotation. However, the present study did not evaluate any computational model. The exploratory AI-oriented video interpretability score should therefore be interpreted as an expert-rated assessment of video characteristics potentially relevant to future workflow-oriented analysis, not as a validated measure of algorithmic performance.

Critical anatomical visualization also varied considerably across videos. Although robotic pelvic surgery theoretically enhances visualization of structures such as the ureter and hypogastric nerves because of stable optics and magnified three-dimensional imaging (2, 21), these structures were not consistently demonstrated. This finding may limit educational interpretability and may be relevant to future studies of AI-oriented anatomical recognition, although actual model performance was not assessed in the present study.

Viewer engagement metrics such as total views and likes were not significantly associated with workflow completeness or AI-oriented interpretability. Similar discrepancies between online popularity and educational reliability have previously been reported in YouTube-based medical education studies (6, 22). These findings suggest that audience engagement may not necessarily reflect structured educational quality. However, the relatively low engagement metrics observed across videos, particularly the low median comment count, may have limited the statistical sensitivity of engagement-related analyses.

The concept of “showcase bias,” defined in the present study as the preferential representation of visually compelling pelvic dissection phases over transitional, reconstructive, and safety-oriented workflow domains, may partially explain several of the observed patterns. In this context, although edited operative videos may improve visual efficiency and audience engagement, excessive fragmentation may reduce procedural continuity and limit suitability for future workflow-oriented video analysis (23). A degree of conceptual circularity should also be acknowledged. Video structure categories were partly based on preservation of operative sequence and continuity, which also contributed to workflow completeness, educational value, and AI-oriented interpretability scores. Therefore, comparisons across video structure categories should not be interpreted as independent validation of the scoring systems. Rather, these comparisons should be considered descriptive analyses showing how different presentation formats aligned with workflow-oriented video characteristics.

From an educational perspective, incomplete preservation of operative workflow continuity may limit the apparent usefulness of publicly available robotic videos for trainee-oriented procedural learning objectives such as operative setup, trocar placement strategy, reconstruction sequencing, and intraoperative safety assessment. Similarly, from a computational perspective, fragmented operative videos lacking continuous phase transitions and stable anatomical representation may be less suitable as candidate material for future AI-oriented studies of surgical phase segmentation, anatomy recognition, procedural annotation, and automated workflow modeling. These findings suggest that many publicly available robotic surgical videos may function more effectively as selective technical demonstrations than as comprehensive procedural datasets suitable for standardized educational or computational workflow analysis.

The present study additionally highlights the growing intersection between robotic colorectal surgery and surgical data science. Surgical video increasingly represents not only an educational resource, but also a structured procedural dataset (24). As AI-assisted surgical technologies continue to evolve, workflow-oriented video standardization and structured surgical video reporting may become increasingly important (11, 25). The present framework was not intended to replace established surgical video assessment systems, but rather to explore workflow-oriented video representation characteristics potentially relevant to robotic surgical education and future AI-oriented applicability. Exploratory workflow-oriented reporting considerations generated from the present study are summarized in Supplementary Table 3. These reporting considerations are author-generated exploratory suggestions derived from the present findings and should not be interpreted as validated consensus recommendations. In addition, the present agreement analysis should be interpreted as an exploratory global assessment framework rather than a formal psychometric validation model.

Several limitations should be acknowledged. First, only publicly accessible YouTube videos were evaluated. Second, the scoring systems used in this study were expert-derived and partially subjective despite predefined operational definitions and consensus review. Third, video editing patterns may have influenced workflow representation independently of actual operative quality. Technical video-engineering parameters such as frame rate, codec structure, watermark overlays, resolution characteristics, and compression-related artifacts were not systematically evaluated and may influence future computational video-analysis applicability. In addition, robotic platform heterogeneity could not be systematically evaluated because platform metadata were incompletely specified in most publicly accessible videos. Differences in robotic systems, optical quality, recording infrastructure, and docking workflows may therefore represent potential confounding factors, particularly for AI-oriented video interpretability assessments. Furthermore, the exploratory AI-oriented video interpretability assessment used in the present study was not validated against an actual computational workflow-recognition algorithm, computer vision model, or automated annotation system. Therefore, the findings should be interpreted as expert-based exploratory observations rather than direct measures of algorithmic performance. The present study also did not evaluate trainee learning outcomes, operative performance, operative quality, patient safety outcomes, or clinical outcomes. Therefore, the findings should be interpreted as describing video-level workflow representation and interpretability rather than downstream educational, clinical, or safety effects. Given the exploratory nature of the study and the relatively limited sample size, subgroup comparisons and multiple statistical associations should be interpreted cautiously. Potential confounders such as video duration, narration, subtitle availability, academic affiliation, channel type, upload year, recording infrastructure, and editing intensity may have influenced composite scores. Because only 39 videos were included and the structured workflow subgroup contained 7 videos, multivariable adjustment was not performed. These subgroup comparisons should therefore be interpreted as exploratory and hypothesis-generating.

Confidence interval reporting for effect-size estimates was not systematically incorporated because of the exploratory design and relatively limited subgroup sizes. In particular, the relatively small number of structured workflow videos may limit the stability of subgroup comparisons and increase susceptibility to subgroup homogeneity effects. Finally, separate weighted agreement analyses or intraclass correlation assessments were not performed for individual workflow, educational, and exploratory AI-oriented scoring domains. In addition, because several workflow-related characteristics may inherently contribute to perceived educational utility, partial conceptual overlap between workflow completeness and educational value domains may exist.

Despite these limitations, this study provides one of the first structured evaluations of robotic LAR videos through the combined perspectives of workflow continuity, educational structure, and exploratory AI-oriented video interpretability assessment rather than relying solely on traditional educational quality metrics.

## Conclusion

Publicly available robotic low anterior resection videos on YouTube predominantly emphasize visually prominent pelvic dissection phases while inconsistently preserving complete operative workflow continuity. Setup-related, reconstructive, and safety-oriented procedural components remain substantially underrepresented. These findings suggest that many publicly available robotic LAR videos may function more as selective technical demonstrations than as standardized workflow-oriented educational resources. Greater emphasis on procedural continuity, anatomical interpretability, reconstruction phases, and safety-oriented workflow representation may improve the clarity and research utility of publicly accessible robotic surgical videos. However, this study did not evaluate trainee learning outcomes, operative performance, patient safety outcomes, or actual AI model performance.

## Data Availability

The raw data supporting the conclusions of this article will be made available by the authors, without undue reservation.
