# Supplementary material for: Beyond surgical showcase: workflow continuity and exploratory AI-oriented video interpretability of robotic low anterior resection videos on YouTube
Source: Front Surg. 2026 Jul 22;13:1886815. doi: 10.3389/fsurg.2026.1886815 (PMC13438161; doi:10.3389/fsurg.2026.1886815)
Supplement: Supplementary file 1 [file Table1.docx]

**YouTube Search Strategy and Video Screening Process**

| Search Term | Approximate Screening Depth | Eligible Videos Identified |
| --- | --- | --- |
| robotic low anterior resection | first 30–40 results | 21 |
| robotic TME | first 30–40 results | 14 |
| robotic LAR rectal cancer | first 20–30 results | 2 |
| robotic rectal cancer surgery | first 20–30 results | 1 |
| robotic colorectal surgery rectum | first 20–30 results | 1 |

Searches were performed between May 1 and May 10, 2026 using an English-language YouTube interface in incognito mode, with a cleared-browser environment and without login-based personalization. No VPN, manual geographic restriction, or location filter was applied. Videos were screened sequentially according to YouTube default relevance-based search output order. Duplicate videos identified across search terms were removed during the screening process. View counts, like counts, and comment counts were recorded on May 10, 2026.
