# Supplementary material for: Beyond surgical showcase: workflow continuity and exploratory AI-oriented video interpretability of robotic low anterior resection videos on YouTube
Source: Front Surg. 2026 Jul 22;13:1886815. doi: 10.3389/fsurg.2026.1886815 (PMC13438161; doi:10.3389/fsurg.2026.1886815)
Supplement: Supplementary file 2 [file Table2.docx]

**Supplementary Table 2. Mapping of Workflow Components to Figure 3 Domain Categories**

| Workflow Domain | Included Components |
| --- | --- |
| Setup | Patient positioning; Trocar placement; Robotic docking |
| Vascular | IMA/IMV dissection |
| Critical Anatomy | Ureter identification; Hypogastric nerve visualization |
| Pelvic/TME | TME dissection; Pelvic dissection; Distal rectal transection |
| Reconstruction | Splenic flexure mobilization; Anastomosis; Specimen extraction |
| Safety | Leak test |

Specimen extraction was included in descriptive domain mapping and heatmap visualization but was not included in the predefined 12-component composite workflow completeness score.
