# Supplementary material for: Beyond surgical showcase: workflow continuity and exploratory AI-oriented video interpretability of robotic low anterior resection videos on YouTube
Source: Front Surg. 2026 Jul 22;13:1886815. doi: 10.3389/fsurg.2026.1886815 (PMC13438161; doi:10.3389/fsurg.2026.1886815)
Supplement: Supplementary file 3 [file Table3.docx]

**Author-Generated Exploratory Workflow-Oriented Reporting Considerations for Publicly Accessible Robotic Surgical Videos**

| Proposed Reporting Item | Purpose |
| --- | --- |
| Procedure indication and operative context | Clarifies surgical indication and procedural background |
| Patient positioning | Improves procedural reproducibility and setup understanding |
| Trocar placement strategy | Facilitates spatial orientation and robotic access planning |
| Robotic docking workflow | Supports workflow continuity and technical standardization |
| Vascular dissection sequence | Enhances procedural phase recognition and educational consistency |
| Critical anatomical landmark visualization | Supports anatomical orientation and AI-oriented recognition tasks |
| Continuous operative phase transitions | Improves workflow continuity and temporal interpretability |
| Reconstruction phase demonstration | Strengthens procedural completeness and trainee-oriented learning |
| Intraoperative safety-oriented steps (e.g., leak test) | Supports procedural safety assessment and workflow integrity |
| Minimal editing-related fragmentation | Preserves temporal continuity for educational and computational workflow analysis |

The proposed reporting considerations were generated as exploratory workflow-oriented recommendations based on the findings of the present study and are not intended to represent validated consensus reporting guidelines.
